# Critical roles of tubular mitochondrial ATP synthase dysfunction in maleic acid-induced acute kidney injury

**DOI:** 10.1007/s10495-023-01897-3

**Published:** 2024-01-28

**Authors:** Hugo Y.-H. Lin, Chan-Jung Liang, Ming-Yu Yang, Phang-Lang Chen, Tzu-Ming Wang, Yen-Hua Chen, Yao-Hsiang Shih, Wangta Liu, Chien-Chih Chiu, Chih-Kang Chiang, Chang-Shen Lin, Han-Chen Lin

**Affiliations:** 1 Internal Medicine, Kaohsiung Municipal Ta-Tung Hospital, Kaohsiung Medical University, Kaohsiung, Taiwan; 2 Division of Nephrology, Department of Internal Medicine, Kaohsiung Medical University Hospital, Kaohsiung Medical University, Kaohsiung, Taiwan; 3 Department of Medicine, College of Medicine, Kaohsiung Medical University, Kaohsiung, Taiwan; 4 Graduate Institute of Medicine, College of Medicine, Kaohsiung Medical University, 100, Shih-Chuan 1St Road, Kaohsiung, 80708 Taiwan; 5 Department of Oral Hygiene, Shu-Zen Junior College of Medicine and Management, Kaohsiung, Taiwan; 6 Grander Pharmacy, Kaohsiung, Taiwan; 7 College of Medicine, Graduate Institute of Clinical Medical Sciences, Chang Gung University, Taoyuan, Taiwan; 8 Department of Biological Chemistry, University of California, Irvine, USA; 9 Department of Medical Research, China Medical University Hospital, Taichung, Taiwan; 10 School of Medicine, Doctoral Program of Clinical and Experimental Medicine, Institute of Biomedical Sciences, College of Medicine, National Sun Yat-Sen University, Kaohsiung, Taiwan; 11 Department of Anatomy, College of Medicine, Kaohsiung Medical University, 100, Shih-Chuan 1St Road, Kaohsiung, 80708 Taiwan; 12 Department of Biotechnology, Kaohsiung Medical University, Kaohsiung, 807 Taiwan; 13 Graduate Institute of Toxicology, National Taiwan University, Taipei, Taiwan; 14 Department of Medical Research, Kaohsiung Medical University Hospital, Kaohsiung, 80756 Taiwan

**Keywords:** AKI, Mitochondria, ATP synthase, Maleic acid

## Abstract

Maleic acid (MA) induces renal tubular cell dysfunction directed to acute kidney injury (AKI). AKI is an increasing global health burden due to its association with mortality and morbidity. However, targeted therapy for AKI is lacking. Previously, we determined mitochondrial-associated proteins are MA-induced AKI affinity proteins. We hypothesized that mitochondrial dysfunction in tubular epithelial cells plays a critical role in AKI. In vivo and in vitro systems have been used to test this hypothesis. For the in vivo model, C57BL/6 mice were intraperitoneally injected with 400 mg/kg body weight MA. For the in vitro model, HK-2 human proximal tubular epithelial cells were treated with 2 mM or 5 mM MA for 24 h. AKI can be induced by administration of MA. In the mice injected with MA, the levels of blood urea nitrogen (BUN) and creatinine in the sera were significantly increased (*p* < 0.005). From the pathological analysis, MA-induced AKI aggravated renal tubular injuries, increased kidney injury molecule-1 (KIM-1) expression and caused renal tubular cell apoptosis. At the cellular level, mitochondrial dysfunction was found with increasing mitochondrial reactive oxygen species (ROS) (*p* < 0.001), uncoupled mitochondrial respiration with decreasing electron transfer system activity (*p* < 0.001), and decreasing ATP production (*p* < 0.05). Under transmission electron microscope (TEM) examination, the cristae formation of mitochondria was defective in MA-induced AKI. To unveil the potential target in mitochondria, gene expression analysis revealed a significantly lower level of ATPase6 (*p* < 0.001). Renal mitochondrial protein levels of ATP subunits 5A1 and 5C1 (*p* < 0.05) were significantly decreased, as confirmed by protein analysis. Our study demonstrated that dysfunction of mitochondria resulting from altered expression of ATP synthase in renal tubular cells is associated with MA-induced AKI. This finding provides a potential novel target to develop new strategies for better prevention and treatment of MA-induced AKI.

**Supplementary Information:**

The online version contains supplementary material available at 10.1007/s10495-023-01897-3.

## Background

MA, which is frequently used as a surfactant or stabilizer in the manufacture of various industrial products [1, 2], has been found in animal models to cause various renal dysfunctions, including type II renal tubular acidosis (RTA II) [1–3], Fanconi's syndrome (FS) [4–7], renal glycosuria, phosphaturia, aminoaciduria, and AKI [8–10]. AKI is an immense clinical problem. The prevalence of AKI is estimated to affect 2–3 people per 1,000 individuals in the United States [11]. There has been an upward trend in the incidence of AKI worldwide over the last decade [12–15]. Although some AKI might be reversible, current evidence indicates that AKI may predispose the kidney to subsequent development of chronic kidney disease (CKD), end-stage renal disease (ESRD) with renal replacement therapy (RRT), or even mortality [14–16]. The mortality associated with AKI is alarmingly high, ranging from 35 to 45% of all patients with a diagnosis of AKI within 90 days after discharge from the hospital [12]. Unfortunately, other than supportive therapy, there is no specific treatment that can be used to effectively prevent or reverse AKI [17].

In our previous study, we discovered MA-induced AKI affinity proteins, which include ATP synthase subunits [18]. These results indicate that mitochondrial dysfunction may be associated with AKI due to MA. Kidneys are one of the most energy-requiring organs in the human body to eliminate uremic toxins, reabsorb essential electrolytes and nutrients through active transport, and maintain systemic hemodynamic balance [19]. The kidney has the second highest mitochondrial content and oxygen consumption after the heart [20, 21]. The mitochondrion is an organelle encapsulated with outer and inner membranes, and the core constituents of mitochondrial respiratory complexes I–V are embedded in the inner membrane. The tricarboxylic acid (TCA) cycle is in the matrix. The respiratory chain creates an electrochemical gradient through the coupled transfer of electrons to oxygen and the transport of protons from the matrix across the inner membrane into the intermembrane space. Mitochondria are the major site of energy production in cells through oxidative phosphorylation and ATP creation [22]. It is not only a powerhouse but also a specialized organelle that regulates cellular metabolism, ROS, apoptosis, and calcium flux [23]. Despite the fact that mitochondrial malfunction has been implicated in a wide variety of pathophysiological changes and human diseases [24], the specific pathophysiological functions of mitochondria in kidney injury are anticipated to be resolved. Moreover, tipping the homeostasis of mitochondria could be a potential target for disease treatment [25].

A better understanding of the molecular mechanisms of MA-induced AKI will allow us to develop specific therapies to correct its mechanistic defects. During AKI, renal proximal tubular epithelial cells are the major site of renal damage and have been implicated in renal injury and recovery [26]. Renal tubular mitochondria dysfunction has been noted during AKI, accompanied by reduced ATP production and increased oxidative stress [27]. Therefore, mitochondria and the signaling pathways modulating oxidative phosphorylation function may become potential targets for therapeutic intervention in AKI. We designed experiments to characterize the role of tubular mitochondria in MA-induced AKI.

## Results

### MA-induced AKI in vivo

**Fig. 1:**
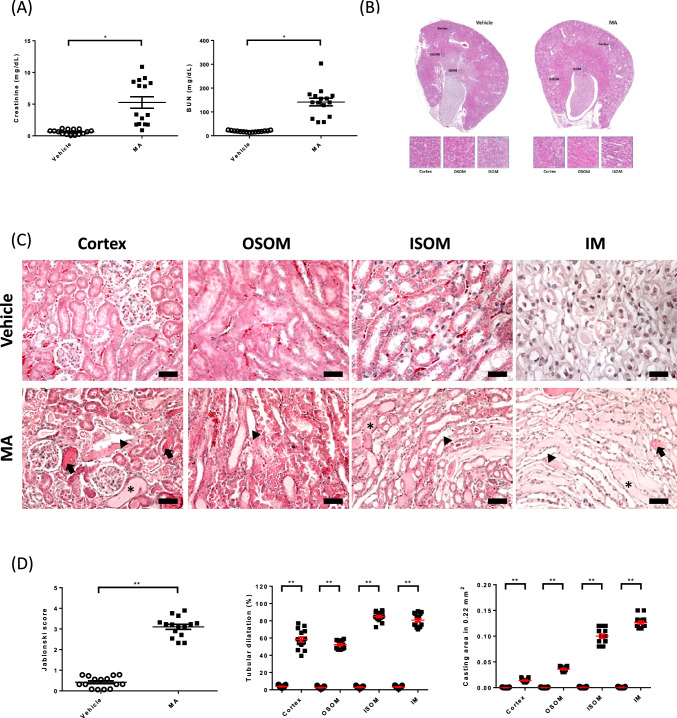
Maleic acid-induced acute kidney injury. After maleic acid (MA) treatment, kidneys were harvested twenty-four hours later. **A** Serum measurements showed an increase in BUN and creatinine (Cr.) after MA treatment. Significantly higher BUN and Cr. was observed in the MA-induced AKI mice (*p < 0.05, **p < 0.001). **B** Renal tissue histology (twenty-four hours after MA) stained with HE to estimate Jabloski scores. OSOM-outer stripe outer medulla. ISOM- inner stripe outer medulla. IM-inner medulla. The Jablonski scores are summarized in the dot graph. **C** Renal injuries were analyzed by HE staining. **D** The dot graph compares the renal tubular injuries between the two groups

The first goal was to investigate whether MA induces AKI in vivo. To this end, C57BL6 mice were injected with MA intraperitoneally. Before treatment, there were no differences in baseline renal function, histology, or Jablonski score between the MA-induced AKI and control groups (Supplementary Fig. 1). Upon MA treatment, both serum BUN and Cr. were significantly higher 24 h later (*p* = 0.0041 and < 0.001 separately, Fig. 1A). To study the changes in renal structure, kidney histology was analyzed. HE staining of renal sections from these mice showed aggravated renal injuries in MA-induced AKI mice (Fig. 1B, C). Jablonski scores of renal tubular injuries were higher (Fig. 1 D, *p* < 0.001) with more tubular brush border loss, tubular lysis, and debris in the tubular lumen space (Fig. 1C). After MA treatment, a significant increase in the ratio of tubular dilatation and casting area was observed (Fig. 1D, p < 0.001, *p* < 0.001). Collectively, these results indicated that MA induced AKI, accompanied by higher serum BUN, creatinine, and renal structure changes.

### MA increased cell death in renal tubule cells in vivo and in vitro

**Fig. 2:**
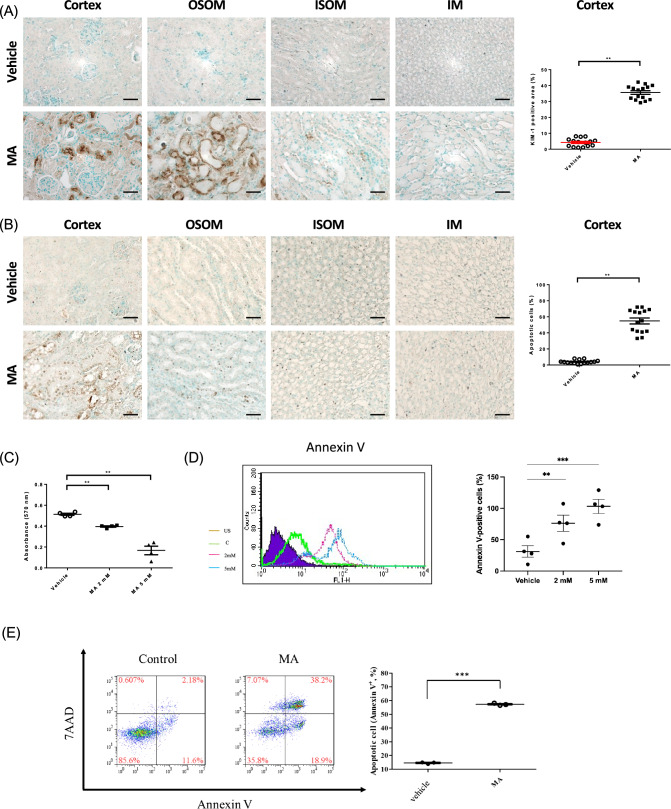
Maleic acid induced renal tubular cell apoptosis. To further investigate the effect of MA on renal tubular cells, renal tissue staining was performed. **A** KIM-1 expression was examined by immunohistochemistry. The dot graph summarizes the results of KIM-1 staining in these mice. **B** Renal apoptosis was analyzed by TUNEL staining. The middle panel shows representative microscopic images, while the right dot graph summarizes the results of the quantification of apoptotic cells from the two groups of mice. **C** Cell viability was assessed by MTT assay. After MA treatment, significantly lower cell viability was observed compared to vehicle treatment. **D** Renal tubular cell apoptosis was analyzed by Annexin V. After MA treatment, a significantly higher signal was observed compared to vehicle. **E** In defining apoptotic cells, after 72 h of exposure to MA (5 mM), the proportion of late apoptotic or necrotic cells rose to 44.2%, while early apoptotic cells increased to 21%

To further gain insight into whether MA-induced AKI was mainly responsible for renal tubular injuries, KIM-1 staining was used (Fig. 2A). KIM-1 was greatly increased in MA-induced AKI, which corroborated more severe renal tubular injuries (*p* < 0.001). To evaluate whether apoptosis was induced by MA in renal tubular cells, TUNEL staining was carried out in renal tissue sections (Fig. 2B). Apoptosis was significantly increased in the renal tubules of MA-induced AKI mice (*p* < 0.001), especially renal proximal tubular cells.

To verify the effect of MA on renal tubular cells and whether there was a dose effect, HK‐2 cells were treated with 2 mM and 5 mM MA. Upon MA treatment, the viability of HK-2 cells decreased (Fig. 2C, p < 0.001 in 2 mM, *p* < 0.001 in 5 mM). A notable decrease in healthy HK-2 cells occurred 24 h after MA treatment (decrease 22.8% in 2 mM, decrease 67.35% in 5 mM, separately at 24 h) compared to the vehicle-treated control group. Annexin V was stained to further assess apoptosis in vitro. As shown in Fig. 2D, HK-2 cells treated with MA (2 and 5 mM) induced a higher apoptosis rate (2 mM, *p* < 0.05; 5 mM, *p* < 0.001). These results suggest that MA aggravates renal injuries by increasing renal tubular cell apoptosis in vivo and in vitro. And MA induces apoptosis in renal tubular epithelial cells through the upregulation of cleaved caspase-3 expression (Supplementary Fig. 2A, C). The ratio of Bax to Bcl2 was also in accordance with the apoptosis (Supplementary Fig. 2B, D).

We stained Annexin V and 7AAD to further define apoptotic cells. In the control group, most cells (85.6%) were identified as healthy, early apoptotic cells accounted for 11.6%, and late apoptotic or necrotic cells constituted 2.18%. These results indicate that most cells in the control group were in a healthy state (Fig. 2E). However, cell death increased when HK-2 cells were treated with MA (5 mM). We observed that after 72 h of exposure to MA, the proportion of late apoptotic or necrotic cells rose to 38.2%, while early apoptotic cells increased to 18.9%.

### Maleic acid modulated cellular respiration in RTE cells

**Fig. 3:**
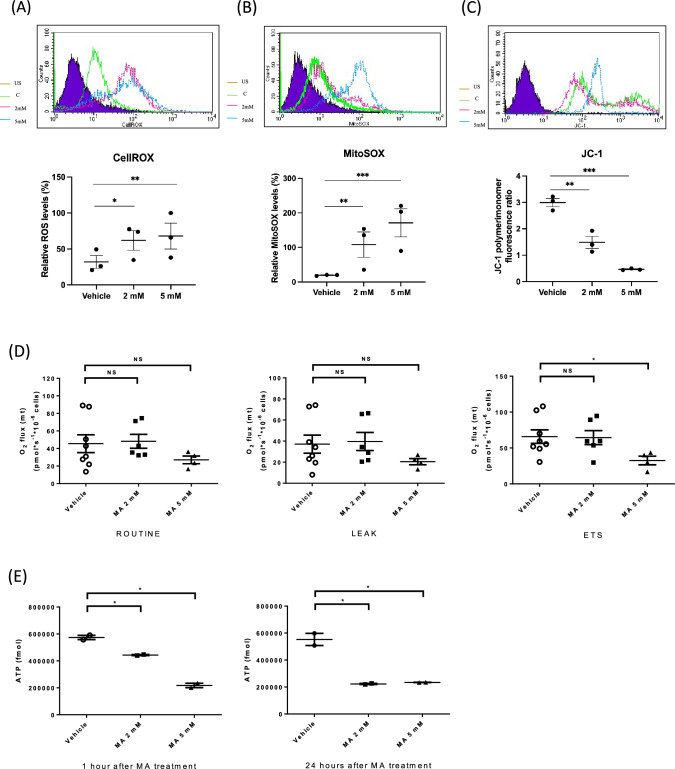
Maleic acid increased cellular oxidative stress and mitochondrial uncoupling, decreased mitochondrial membrane potential, and decreased cellular ATP in renal tubular epithelial cells. To investigate the effect of MA on the mitochondria of renal tubular cells, renal tubular cell staining with flow cytometry was performed. **A** CellROX expression was examined by flow cytometry. The bar graph summarizes the results of CellROX staining in these HK-2 cells. **B** MitoSOX expression was examined by flaw cytometry. The dot graph summarizes the results of quantification from the two groups of cells. **C** JC-1 was assessed by flow cytometry. After MA treatment, significantly lower mitochondrial potential was observed compared to vehicle treatment. **D** HK-2 cells were plated for OROBORUS analysis. After basal extracellular respiration rate (O2 flux) analysis, different inhibitors were injected sequentially to measure different stages of respiration (complex V inhibitor: oligomycin, uncoupler: FCCP, and complex I inhibitor: rotenone.) as shown. Although ROUTINE respiration and LEAK respiration were not significantly different between MA-treated cells and vehicle-treated cells, there was a significantly lower ETS of MA-treated cells than vehicle-treated cells. **E** One hour after MA treatment, ATP levels were reduced in 5 mM MA-treated cells. Twenty-four hours after MA treatment, ATP levels were significantly lower in 2 mM and 5 mM treated cells

The next series of experiments were designed to investigate whether MA-induced AKI influences mitochondrial function. To this end, 25 μM Cell-Rox and 5 μM MitoSox were stained to evaluate the oxidative stress of renal tubular cells after MA treatment. We observed that MA (2 and 5 mM) treatment significantly increased the oxidative stress of HK-2 cells (*p* < 0.05 and *p* < 0.001, Fig. 3A). We compared mitochondrial oxidative stress in MA-treated and vehicle-treated HK-2 cells, and mitochondrial oxidative stress was significantly increased in MA-treated HK-2 cells, as expected (2 mM, *p* < 0.05 and 5 mM *p* < 0.001, Fig. 3B). To further study whether MA affects mitochondrial membrane potential, JC-1 5 μM staining with flow cytometry analysis was carried out. As shown in Fig. 3C, we discovered that MA (2 and 5 mM) treatment significantly decreased the mitochondrial membrane potential of HK-2 cells (*p* < 0.005, *p* < 0.001).

To analyze how MA modulates HK-2 cellular bioenergetics, we used Oxygraph-2k (O2k, OROBOROS Instruments, Innsbruck, Austria), a cellular respirometer, to assess mitochondrial function. Routine respiration represents measurements of cell respiration during physiological activity. Leak respiration indicates that respiration compensates for proton leakage, proton slip and cation cycling. The electron transfer system (ETS) capacity evaluates the open-circuit operation of the transmembrane proton gradient. Overall, the routine respiration of the MA-treated HK-2 cells (2 and 5 mM) was not significantly different from that of the control cells (Figure 3D). The leak respiration profile of the MA-treated HK-2 cells was not significantly different from that of the control cells. However, we noticed that the ETS profile of the MA-treated HK-2 cells (5 mM) was significantly lower than that of the vehicle-treated cells (*p *< 0.05). This result indicated impaired oxidative phosphorylation with higher oxidative stress. ATP production was similarly reduced in renal tubular cells 1 h (2 mM, *p *= 0.0150, and 5 mM, *p *= 0.0041) and 24 h (2 mM, *p *= 0.0181, and 5 mM, *p *= 0.0193) after MA treatment (Figure 3E). These findings suggest that MA caused mitochondrial dysfunction with a reduced electrochemical gradient, which ultimately led to diminished ATP production.

### MA altered the mitochondrial ultrastructure of renal tubular cells

**Fig. 4:**
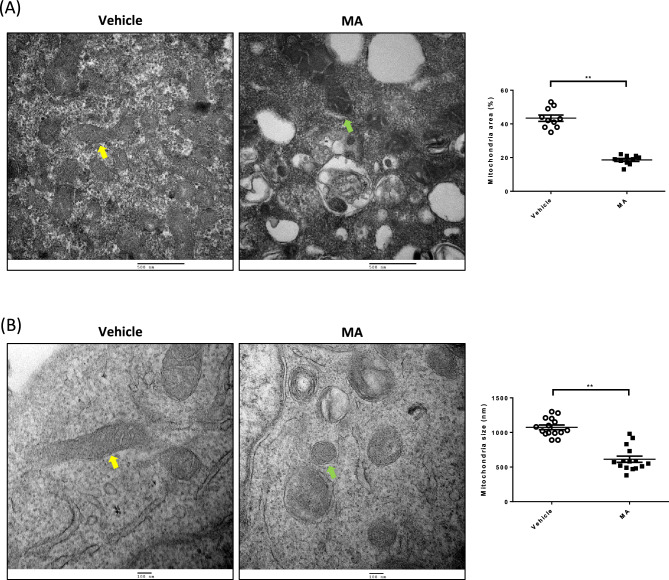
Maleic acid disrupts mitochondrial crista formation. To investigate the effect of mitochondrial ultrastructure after MA treatment on renal tubular cells, a transelectron microscope was used. **A** In MA-treated HK-2 cells, swollen, homogenized, and whirled cristae were observed, corresponding to mitochondrial damage (green arrow), whereas the mitochondrial structure in the control groups was normal (yellow arrow). The dot graph summarizes the quantification of the total numerical density of mitochondria between the two groups. Mitochondrial size between the two groups of HK-2 cells. **B** At a higher scale, damaged mitochondria with disrupted and discontinuous outer membranes and deficient cristae were observed in MA-treated HK-2 cells (green arrow). The mitochondrial sizes were significantly decreased in MA-treated HK-2 cells (Color figure online)

To characterize morphometric analysis of MA in renal tubular cells, mitochondrial ultrastructures were examined with TEM. HK-2 cells treated with MA showed disruption of the mitochondrial distribution compared with vehicle-treated HK-2 cells (Fig. 4A). Morphometric analysis revealed a decreased mitochondrial area ratio (18.6 ± 0.86% mitochondria per field in vehicle-treated versus 43.4 ± 1.88% in MA-treated) (*p* < 0.001) (Fig. 4A). Furthermore, there was a significant decrease in the size of mitochondria (Fig. 4B, green arrow) in MA-treated HK-2 cells compared with vehicle-treated HK-2 cells (Fig. 4B, yellow arrow) (*p* < 0.001). In MA-treated HK-2 cells, swollen, homogenized, and whirled cristae were observed (Fig. 4B, green arrow), whereas the mitochondrial structure in the vehicle-treated groups was normal (Fig. 4B, yellow arrow). These findings suggest that MA triggered mitochondrial deformation with cristae disorganization.

### Decreased ATP synthase subunits α and γ in MA-induced AKI

**Fig. 5:**
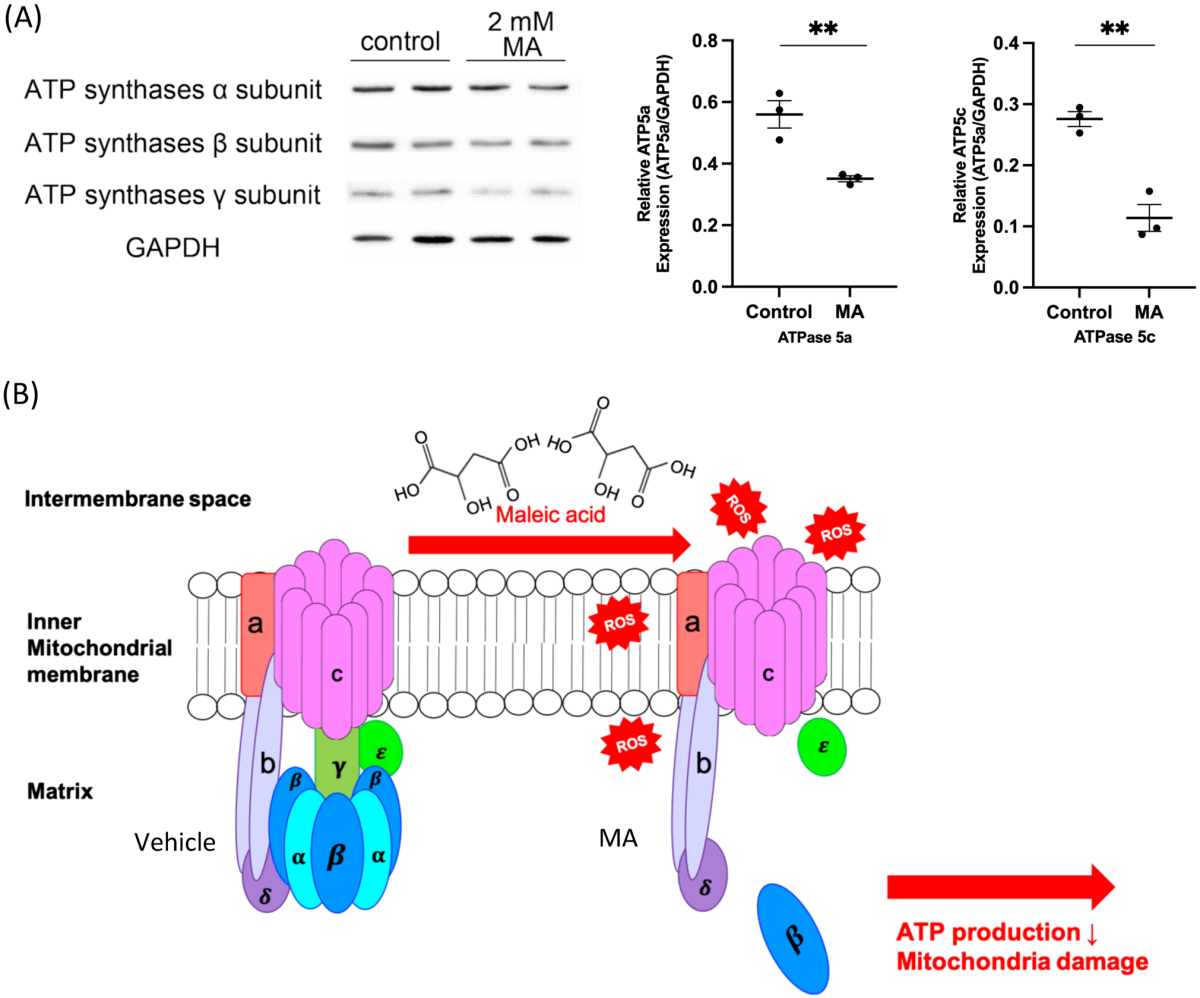
MA decreases ATP synthase alpha and gamma. To uncover the intramitochondrial targets of MA-induced AKI, gene expression analysis was carried out. There was significantly lower expression of ATP synthase in MA-treated HK-2 cells. To quantify the protein expression of ATP synthase, the mitochondrial fraction was isolated, and the expression of ATP synthase protein was analyzed in vivo and in vitro. **A** ATP synthase subunits alpha and gamma were expressed at significantly lower levels in MA-treated HK-2 cells than in vehicle-treated cells in vitro. **B** In the schematic diagram of the mechanism of MA-induced AKI, the tubular mitochondrial ATP synthase complex was defective. These effects deformed mitochondrial crista formation and led to decreased ATP generation and electron transport systems and increased reactive oxidative species production

To explore the molecular mechanism of mitochondria in MA-induced AKI, we executed next-generation sequencing of renal tubular cells treated with MA and vehicle. The mRNA expression levels of NADH dehydrogenase (ubiquinone), including MT-ND1, MT-ND2, and MT-ND3, were significantly decreased in MA-treated HK-2 cells compared with vehicle-treated cells (*p* < 0.005) (Table 1). Upon MA treatment, the mRNA expression of UQCR11, which oversees the coding ubiquinol-cytochrome c reductase complex (complex III), was notably reduced (*p* < 0.05). A significant decrease in MT-CO3, which encodes cytochrome c oxidase III, was diminished compared to vehicle treatment (*p* < 0.001). The mRNA expression of MT-ATP6, which encodes Fo subunit 6 of ATP synthase, was substantially decreased in HK-2 cells treated with MA compared with that in cells treated with vehicle (*p* < 0.001). These results may explain the previous findings of respirometry. MA affects the electron transfer system of mitochondria and ultimately reduces ATP production. To validate the protein expression of ATP synthase in MA-induced AKI, we isolated and analyzed ATP synthase. The decreased expression of ATP synthase subunits α and γ was corroborated in MA-induced AKI (*p* < 0.05) (Fig. 5A). These results collectively indicated that MA targeted ATP synthase and may disrupt the ATP synthase complex (Fig. 5B).

**Table 1:** Analysis of target gene decreased expression in MA from next generation sequencing

Gene	Function	mRNA fold change	P value
MT-ND1	Mitochondrially encoded NADH:ubiquinone oxidoreductase core subunit 1	− 1.9296	< 0.001
MT-ND2	Mitochondrially encoded NADH:ubiquinone Oxidoreductase core subunit 2	− 2.2519	< 0.001
MT-ND3	Mitochondrially encoded NADH:ubiquinone Oxidoreductase core subunit 2	− 2.364	< 0.001
UQCR11	Ubiquinol-cytochrome c reductase, complex III subunit XI	− 1.3631	0.00181
MT-ATP6	Mitochondrially encoded ATP synthase 6	− 2.5215	< 0.001
MT-CO2	Mitochondrially encoded cytochrome c oxidase III	− 2.8097	< 0.001

## Discussion

This study illustrated that MA led to AKI with renal proximal tubular injuries. In renal tubular cells, MA interacted with mitochondria, resulting in increased mitochondrial oxidative stress, decreased ETS, decreased mitochondrial membrane potential, and reduced ATP production. MA caused the diminution of ATP synthase subunits α and γ, resulting in a defect in the ATP synthase complex. The ultrastructure of mitochondria was also changed. Therefore, ATP synthase is a novel target of MA-induced AKI, which may represent a potential therapeutic objective.

### MA and kidney

MA-induced kidney injuries are histologically characterized by ischemic and swollen kidneys with apical vacuolization, lumen dilatation of proximal tubules, and interstitial edema [28]. MA has been widely reported and utilized as an AKI inducer in various experimental studies. And in Taiwan, however, there have been instances in which some food manufacturers have unscrupulously added MA as an emulsifier to their starch-based food products, including frozen dumplings and glutinous desserts [18]. It has demonstrated its capacity to induce renal injury by causing tubular damage, inflammation, and impaired renal function, thereby reflecting the pathophysiological features observed in clinical AKI. Several studies have demonstrated that MA nephropathy includes dose-dependent glycosuria, phosphaturia, and aminoaciduria [8]. In the study of Schärer et al., MA inhibited renal gluconeogenesis much more strongly with the substrates pyruvate, lactate, and α-ketoglutanate than glycerol, succinate, fumarate, and malate [29]. This result indicated that MA may interfere with pyruvate carboxylase, which catalyzes the conversion of pyruvic acid into oxaloacetate. In addition to gluconeogenesis, impaired ATP synthesis has also been identified in MA-induced kidney injuries [30]. Based on an in vivo model, MA provoked Fanconi syndrome associated with inhibition of Na–K-ATPase and defective production of ATP. In a study using alpha-methyl-D-glucoside uptake, MA impaired glucose transport in renal proximal tubules by inhibiting D-glucose movement from the cytoplasm across the antiluminal membrane into the blood and stimulated movement back across the brush-border membrane into urine [31]. A study measured and compared isolated mitochondria from the kidney and liver with and without MA treatment [32]. With MA, the CoA-SH content decreased as low as 10% of the original. The acid-soluble acyl-CoA also decreased to approximately 70% of the initial value. These results revealed that MA may interfere with renal proximal mitochondrial function. In the study of Tapia et al., MA-induced kidney injuries not only induced ROS production, shortened oxygen consumption in ADP-stimulated mitochondria and reduced the respiratory control index when using malate/glutamate as a substrate [33]. By using curcumin, MA-induced kidney injuries could be prevented by preserving mitochondrial oxygen consumption and respiratory complex I activity. This study demonstrates that mitochondrial targeting has potential for MA-induced kidney injury therapy.

### The role of mitochondria in renal tubule AKI

AKI is characterized by acute tubular cell necrosis, inflammatory responses, and vascular dysfunction [34]. Injury and death of tubular cells have been recognized as key elements in the development of AKI. With the renal tubular apoptosis induced by AKI, the activation of the intrinsic pathways of apoptosis has been unveiled in both in vitro and in vivo models [35]. The kidney is one of the most energy-requiring organs in the human body [19]. A greater number of renal mitochondria reside in the renal proximal tubule [36]. Mitochondria act as crucial cellular organelles for ATP production [22]. Since ATP propels most biochemical reactions in cells, defective mitochondrial bioenergetics may disrupt important biological processes. In addition to energy production, mitochondrial signaling pathways and downstream calcium flux indicate its vital role in cellular metabolism, ROS production, and apoptosis [23]. As demonstrated in this study, MA-induced AKI impaired the mitochondrial function of renal tubular cells not only by decreasing membrane potential and ETS and reducing ATP production but also by increasing ROS and ultimately leading to renal tubular cell apoptosis. Apoptosis has been widely recognized as a key mechanism contributing to renal tubular injury in AKI. Numerous studies have demonstrated the prominent role of apoptosis in tubular cell death during the development of AKI [37]. Mitochondrial injury is the center of renal tubular cell apoptosis in AKI. The loss of the mitochondrial cross-membrane electrochemical gradient is regarded as an essential control point that triggers apoptosis [38]. MA-induced AKI, similar to ischemia‒reperfusion injury AKI, causes mitochondrial leakage of renal tubular cells and probably represents a point of no return in the life and death decision during apoptosis [39].

### Oxidative stress in AKI

Oxidative stress is a common feature of AKI. It results from the disruption of the redox system marked by a notable overproduction of ROS. ROS occur before the increase in serum BUN and Cr. level and is an early pathologic event in AKI [40]. In addition to acute production, oxidative stress in AKI may adversely affect renal recovery since overproduction of ROS contributes to renal fibrosis [41, 42]. Mitochondria are one of the major sources of ROS production. NADH-ubiquinone oxidoreductase (complex I) and ubiquinol-cytochrome c reductase (complex III) transfer electrons to O_2_ to produce O_2_- and generate H_2_O_2_ by reacting with manganese superoxide dismutase (MnSOD) [43]. Our study results are in accordance with previous research showing that MA-induced AKI increases ROS production with the major source being mitochondria [44]. MA diminishes the mRNA production of complexes I (MT-ND1, 2, 3), III (UQCR11, MT-CO2), and V (MT-ATP6), combined with the reduction of ETS, and ultimately leads to the overproduction of ROS.

### Bioenergetics with mitochondrial electron transport and membrane potential

The ETC is a series of complexes that transfer electrons from electron donors to electron receptors via redox reactions and couples this electron transfer with the transfer of protons across the membrane. The ETC is the driver of oxidative phosphorylation and is the target of many nephrotoxic medications [45, 46]. With the damage of complexes by MA, the opening of the permeability transition pore will lead to dissipation of the mitochondrial membrane potential. Our data suggested that MA decreases mitochondrial membrane potential by reducing ETS.

### Cristae formation of mitochondria

TEM demonstrated the changes in cytoarchitectonic structures after MA treatment of renal tubular cells. These ultrastructural alterations were generally characterized by alterations in the sizes of mitochondria and cristae disorganization, all of which have significant consequences for maintaining renal tubular physiological function. We noted a > 50% reduction in the mitochondrial area ratio, which destroyed the maintenance of mitochondrial cristae in renal tubular cells. Similarly, MA exhibited Type III and IV mitochondria of renal tubular cells that are indicative of damaged mitochondria. In contrast, such mitochondrial alterations were not observed in vehicle treatment. These changes in the oxidative phosphorylation system may have contributed to the occurrence of renal tubular dysfunction after AKI. The disruption of mitochondrial structure should reflect the inhibition of oxidative phosphorylation and the formation of ATP. Thus, a deficit in mitochondrial structure and energy production appears to be an important determinant of MA-induced AKI.

### ATP synthase

During cell stress and apoptosis, ATP production is the fuel for cell resistance to cell stress. ATP production estimates showed that mitochondrial energy production at 24 h after MA injection was reduced by 60%, indicating that energy production by the mitochondria was inadequate to accommodate AKI and increased renal energy demand. Our NGS evidence also suggests that MA results in decreased gene expression of complexes I-V. Finally, we highlight the documented effects observed in our isolated mitochondrial assays, suggesting that ATP synthase (complex V) is one of the main targets of MA. ATP synthases are important proteins that regenerate molecular fuel [47]. It is composed of 18 protein subunits and is also known as complex V. Transmembrane proton motives (Δp) are used as an energy source for ATP synthase. Δp drives a mechanical rotation mechanism through a portion of ATP synthase (Fo). Fo is linked to F1, which is the catalytic part of the enzyme complex where ATP is formed from ADP and Pi. The F_1_ domain contains three noncatalytic α- and three catalytic β-subunits that alternate in a hexameric structure around the central stalk. Part of the γ-subunit is completely enveloped in this α3β3 domain and occupies its central axis attaching to the Fo domain[48]. Beyond oxidative phosphorylation, ATP synthase is involved in the morphology of mitochondria by contributing to the generation of inner membrane cristae [49]. Two monomers of ATP synthase are joined together, and some subunit–subunit interactions are involved in inner membrane bending, which is essential for cristae formation [50–52]. Our study indicated that MA decreases the expression of ATP synthase subunits α and γ. These results may not only induce malfunction of the ATP synthase complex but also ultimately lead to cristae disorganization inside mitochondria.

## Conclusions

The identification of mitochondrial ATP synthase in the pathogenesis of AKI has shifted the paradigm of kidney injury to new attention to the pathological role of the proximal tubule in AKI. Our results suggest that mitochondrial ATP synthase indirectly leads to the development of consequent renal failure by maintaining the integrity of mitochondrial structure and function. The data presented in this study help fill an important knowledge gap on the role of proximal renal tubule mitochondria in AKI and its subsequent renal failure. The new mechanism of mitochondrial injury may represent a novel target to develop new strategies for better prevention and treatment of AKI.

## Methods

### Materials

ATP5a1 and ATP5c1 antibodies (14676-1-AP and 10910-1-AP, respectively) were purchased from Proteintech (Planegg-Martinsried, Germany). ATP5B antibody (HPA001528) was purchased from Sigma (St. Louis, Missouri). Caspase 3/p17/p19 antibody (19677-1-AP) was purchased from Proteintech (Rosemont, Illinois). Bcl-2 Polyclonal antibody (E-AB-60012) and Bax Polyclonal antibody (E-AB-66898) were purchased from Elabscience (Houston, Texas). The QuantiChromTM Urea Assay Kit and QuantiChromTM Creatinine Assay Kit were purchased from BioAssay Systems (Hayward, CA). Chemicals and reagents were from Sigma Aldrich (St Louis, MO).

### In vivo model of MA-induced AKI

All animal work was performed in an Association for Assessment and Accreditation of Laboratory Animal Care International (AAALAC)-accredited facility. The experimental protocol was approved by the Institutional Animal Care and Use Committee at Kaohsiung Medical University and complied with the National Institutes of Health guidelines. Eight-week-old C57BL/6 J mice (Jackson Laboratory, Bar Harbor, ME) were used to study the effect of MA-induced AKI. The MA injection was carried out at 400 mg/kg body weight, and the mice were sacrificed 24 h after injection. Mice were kept at ambient temperature (30 °C–32 °C) after MA injection. Control mice were subjected to the same procedure (vehicle).

### Serum BUN and Cr

At the end of the experiment, blood was collected from the retro-orbital plexus under anesthesia with 2.5% tribromoethanol (avertin). Serum was obtained by centrifugation at 10,000 relative centrifuge force (RCF) for 30 min at room temperature. Serum BUN and Cr. was determined with a colorimetric assay kit (BioAssay Systems, Hayward, CA) and analyzed with a Biotek Synergy HT plate reader.

### Histology and immunohistochemistry (IHC)

Kidney samples were fixed in 10% formalin for 24 h, dehydrated, and embedded in paraffin. Paraffin-embedded kidney blocks were sectioned at 4 μm thickness. Hematoxylin and eosin staining was performed to assess general morphology and renal structure injury. Renal damage in proximal tubules from the cortex area and outer stripe of the outer medulla (OSOM) of the kidney was evaluated with a semiquantitative analysis of histologically damaged areas as previously described [53]. The Jablonski grading scale (0–4) was used for the assessment of AKI-induced necrosis of the overall proximal tubules [54]. To visualize kidney injury molecule-1 (KMI-1) expression by IHC staining, sections were incubated with KIM-1 antibody (R&D Systems) and biotinylated α-rabbit secondary antibody (Vector Lab) for 45 min at room temperature. The slides were counterstained with 1% methyl green. At least 20 microscopic fields were randomly selected from each tissue section, and the percentage of positively stained tubules was assessed by ImageJ for quantitative analysis. The investigators who scored the histology images were blinded to the samples.

### TUNEL stain

Kidney sections were deparaffinized and incubated with 0.05% saponin at room temperature for antigen unmasking. To identify renal cell apoptosis, renal sections were stained with the In Situ Cell Death Detection Kit according to the manufacturer’s instructions (Roche, Basel, Switzerland). At the end of the staining, the slides were counterstained with 1% methyl green at the end of staining, and the images were acquired with a Zeiss AxioPlan2 microscope. At least 20 microscopic fields were randomly selected from each tissue section, and TUNEL-positive nuclei were analyzed with ImageJ for quantitative analysis.

### In vitro model of MA-induced AKI

HK2 cells (human kidney proximal tubular cells) were purchased from American Type Culture Collection (ATCC, Manassas, VA, USA). HK2 cells were cultured in commercial keratinocyte medium containing 10% fetal bovine serum (FBS; HyClone), 2 mM glutamine, 100 U/ml penicillin and 100 mg/ml streptomycin in a humidified atmosphere with 5% CO2 at 37 °C. In preparation for treatment of MA (Sigma, St. Louis, MO, USA), cells were seeded in clear-bottom 96-well plates (Greiner, Frienckenhausen, Germany) at a density of 1 × 10^5^. Serially diluted concentrations of MA were prepared in MA in HK-2 media. All treatments were added to appropriate wells for the intended exposure duration and kept at 37 °C in a humidified incubator with 5% CO2 before performing experiments. For the in vitro study, HK2 cells were induced with MA at 2 mM and 5 mM for 24 h separately. To evaluate the cytotoxic effect of MA, the MTT assay was used to measure cell viability in MA-treated and vehicle-treated HK-2 cells.

### Apoptosis, ROS production, and mitochondrial membrane potential

To identify MA-induced apoptosis, the apoptotic cells were identified by Annexin V (Alexa Fluor conjugate) assay. The cells were treated with MA (2 and 5 mM) or without and stained with Annexin V buffer according to the user guidebook (Thermo Fisher Scientific). Flow cytometric analyses were performed according to recommendations of the International Society for Advancement of Cytometry with a CytoFlex (Beckman Coulter, Fullerton, CA, USA) [55]. For apoptotic cell definition, the Annexin V-FITC/PI Apoptosis Detection Kit (Strong Biotech Corporation, Taipei, Taiwan) was utilized. For measurement of ROS production in response to MA, the cells were treated with MA, followed by the addition of CellRox (25 μM) or MitoSox (5 μM). For measurement of mitochondrial membrane potential in response to MA, the cells were treated with MA, followed by the addition of JC-1 (5 μM).

### Mitochondrial oxygen consumption

The oxygen consumption rate (OCR) and the rate of mitochondrial hydrogen peroxide production were simultaneously determined using Oxygraph-2 k (O2k, OROBOROS Instruments, Innsbruck, Austria) by a previously described method [56]. The cells were cultured in 10 cm dishes and treated with MA for 24 h. After trypsinization, cells (2 × 10^6^) were collected and subjected to mitochondrial oxygen consumption measurement. The protocol for mitochondrial oxygen consumption measurement was described previously [57]. After measurement of baseline, the following reagents were added: oligomycin (0.2 µg/ml; ATP synthase inhibitor), carbonyl cyanide-p-trifluoromethoxyphenylhydrazone (2 µM; uncoupling agent), retenone (1 µM; inhibitor of electron transport system), and antimycin (1 µM; inhibitor of electron transport system) [58]. The capacity of mitochondrial respiration was calculated as the value derived from the uncoupling agent and baseline.

### ATP production assay

For measurement of ATP production in response to MA, an ATP Detection Assay Kit (#700,410) (Cayman Chemical, Ann Arbor) was used. The cells were cultured and incubated with MA for 1 h and 24 h, followed by the addition of 1X ATP detection buffer following the guidelines of the manufacturer. After washing 3 times with PBS, the cells were trypsinized and collected by centrifugation at 750 g. The luminescence was measured after adding D-Luciferin Solution and Luciferase by a Synergy HT multidetection microplate reader.

### TEM

HK-2 cells were treated with MA and vehicle separately. The cells were digested and collected routinely. The cells were suspended and fixed with 2.5% glutaraldehyde in 0.1 mM PBS (pH 7.4) at 4 °C for 2 h. After washing twice with PBS, the cells were treated with conventional dehydration, osmosis, embedding, sectioning, and staining. The ultrastructure of the cells was observed under a Hitachi H7700 electron microscope.

### Mitochondria preparation

Renal cortical and outer medulla tissues were isolated from the kidney, minced, washed with ice-cold PBS 3 times, and suspended in mitochondria isolation buffer (20 mM HEPES–KOH, pH 7.2, 10 mM KCl, 1.5 mM MgCl_2_, 1.0 mM sodium EDTA, 1.0 mM sodium EGTA, 1.0 mM dithiothreitol, 2 mM phenylmethylsulfonyl fluoride, 20 mM NaF, 2 mM Na3VO4, and 250 mM sucrose). After centrifugation at 1750 RPG for 10 min at 4 °C, the samples were incubated on ice for 30 min and homogenized with 20 strokes of a loose pestle and 50 strokes of a tight pestle in a Dounce homogenizer. The nuclei and cell debris were removed by centrifugation at 1000×*g* for 15 min at 4 °C. The supernatants were centrifuged at 10,000×*g* for 30 min at 4 °C, and the resulting mitochondrial fractions were resuspended in mitochondria isolation buffer. The supernatants were further centrifuged at 100,000×*g* for 1 h at 4 °C. The supernatants and mitochondrial fractions were stored at − 80 °C if not immediately used for biochemical analysis.

### Western blots

For the in vivo model, the mitochondrial fractions were dissolved in 2% lauryl maltoside solution supplemented with 10% Sigma FAST™ protease inhibitor (Sigma‒Aldrich, S8820). Protein contents were determined with an Eppendorf Bio Photometer by the BCA method [59]. Equal amounts of proteins from each sample were resolved with a 10% SDS-polyacrylamide gel and then transferred onto polyvinylidene difluoride membranes. For the in vitro model, HK-2 cells were treated with either MA (5 mM) or vehicle (control). Following treatment, cell lysates were subjected to Western blot analysis. The blots were probed with an antibody against cleaved caspase-3. The images were acquired with a KETA CX chemiluminescence system (Wealtec Corp., USA) and analyzed with ImageJ.

### Next-generation sequencing

Total RNA was extracted using TRIzol® Reagent (Thermo Fisher Scientific, Waltham, MA, USA, Catalog No. 15596018) according to the instruction manual. The purified RNAs were quantified at OD260 nm using an ND-1000 spectrophotometer (NanoDrop Technologies, Wilmington, DE, USA) and qualitatively analyzed using a Bioanalyzer 2100 (Agilent Technologies, Santa Clara, CA, USA) with an RNA 6000 LabChip kit (Agilent Technologies, Santa Clara, CA, USA). Library preparation and deep sequencing were carried out by an Illumina NovaSeq6000 sequencer at Tools (Taiwan). Expression analysis was performed and analyzed by Novogene (Tools, Taiwan).

### Statistical analysis

Data are presented as the mean ± SD, unless noted otherwise. Statistical data were analyzed with GraphPad Prism 5 software, with Student’s t test or ANOVA when indicated. The region of interest (ROI) of the western blot was quantified by ImageJ, normalized to the ROI value of the loading control and analyzed with Student’s t test. The statistical significance level was set at *p* < 0.05.

### Supplementary Information

Below is the link to the electronic supplementary material.

Supplementary file1 (PDF 538 kb)

Supplementary file2 (DOCX 12 kb)

## Data Availability

The data that support the findings of this study are available from the corresponding author, HL, upon reasonable request.
