# Supplementary material for: Critical roles of tubular mitochondrial ATP synthase dysfunction in maleic acid-induced acute kidney injury
Source: Apoptosis. 2024 Jan 28;29(5-6):620–34. doi: 10.1007/s10495-023-01897-3 (PMC11055741; doi:10.1007/s10495-023-01897-3)

Supplement Figure 1: Baseline Characteristics of Vehicle and MA groups

(A)

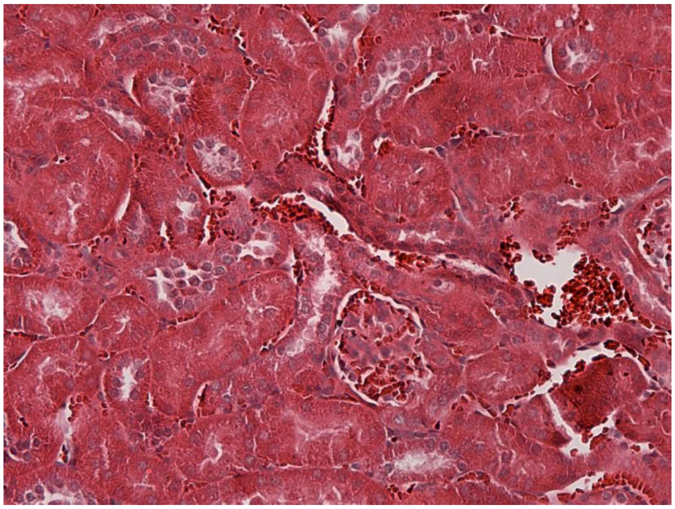

(C)

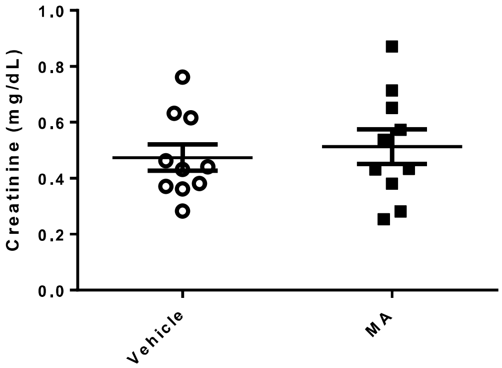

(B)

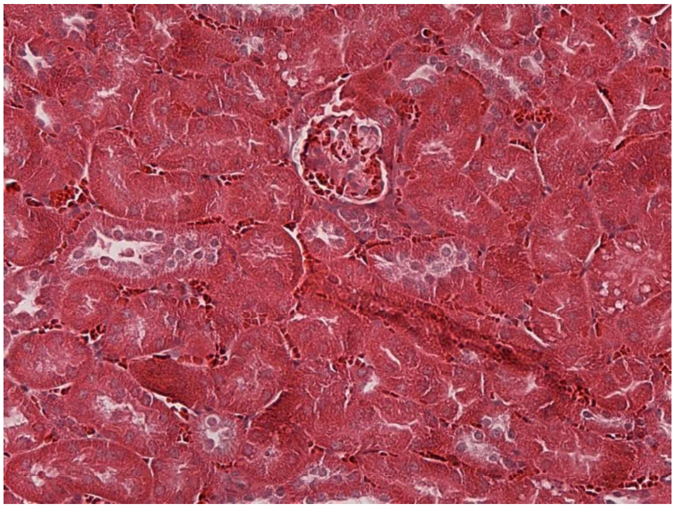

(D)

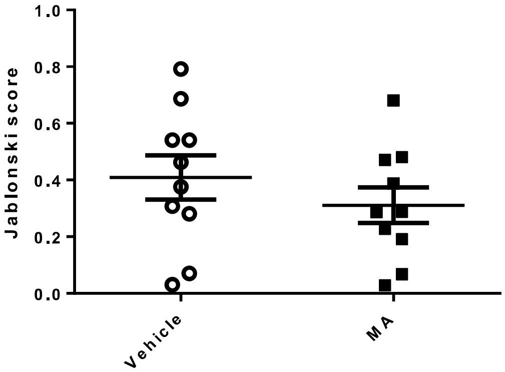

Supplement Figure 2: MA induces RTE apoptosis by the upregulation of caspase-3 expression

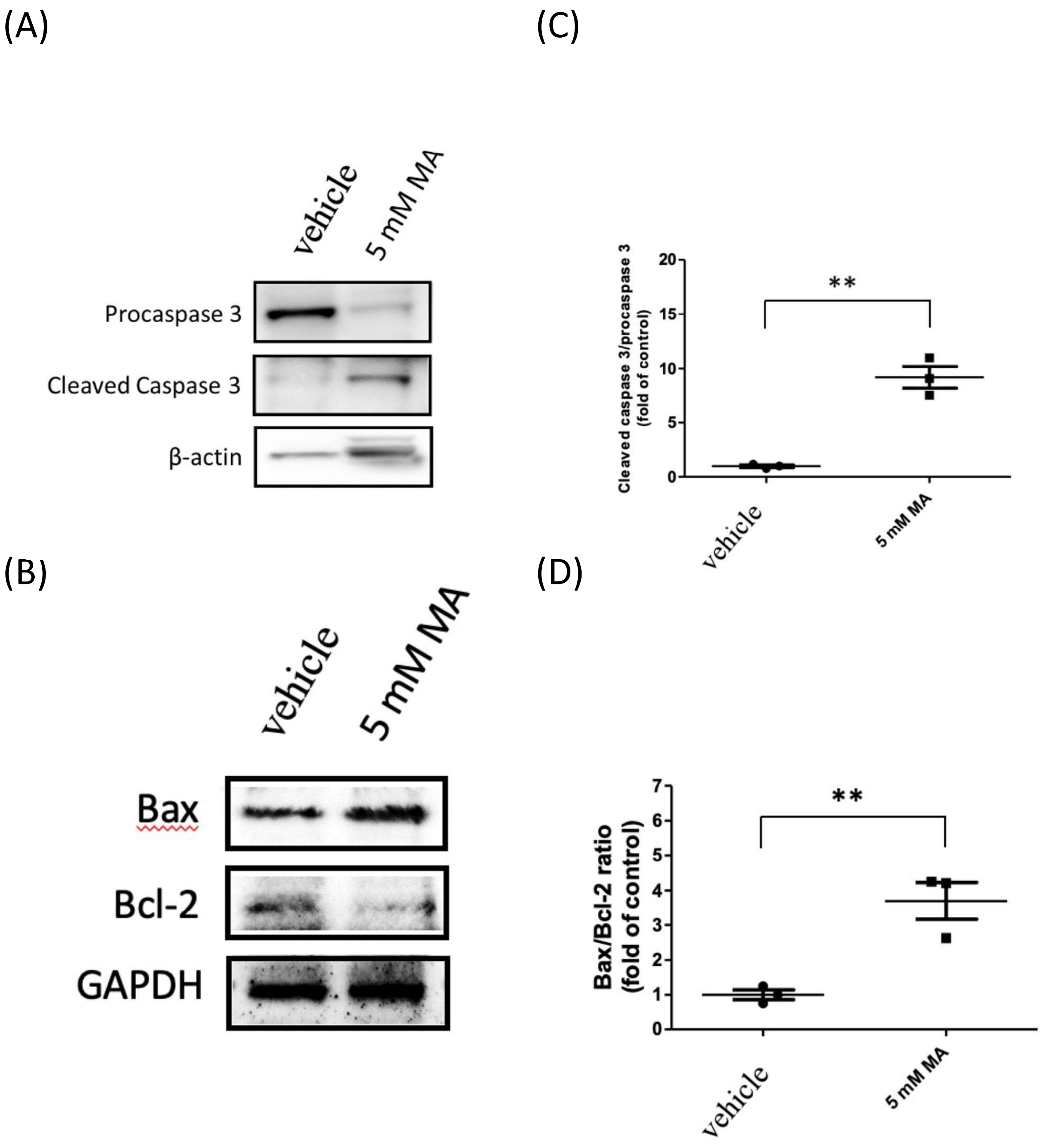

Supplement: Supplementary file 1 — Supplementary file1 (PDF 538 kb) [file 10495_2023_1897_MOESM1_ESM.pdf]
