# Supplementary material for: Critical roles of tubular mitochondrial ATP synthase dysfunction in maleic acid-induced acute kidney injury
Source: Apoptosis. 2024 Jan 28;29(5-6):620–34. doi: 10.1007/s10495-023-01897-3 (PMC11055741; doi:10.1007/s10495-023-01897-3)
Supplement: Supplementary file 2 — Supplementary file2 (DOCX 12 kb) [file 10495_2023_1897_MOESM2_ESM.docx]

Supplementary Figure Legend

**Supplementary Figure 1.**

Before maleic acid (MA) treatment, kidneys were harvested. Renal tissue histology stained with HE to estimate Jabloski scores. In representative sections, there were no significant differences in morphology between the (A) MA and (B) vehicle groups before treatment. (C) Serum measurements showed that there were no significant differences in creatinine (Cr.) in both groups of mice before MA and vehicle treatment. (D) The differences in Jabloski scores between the two groups were not significant.

**Supplementary Figure 2.**

Renal tubular epithelial cells were treated with either maleic acid (5 mM) or vehicle (control). Following treatment, cell lysates were subjected to Western blot analysis. The blots were probed with an antibody against cleaved caspase-3, BAX, and Bcl-2. The results revealed a significant increase in the expression of (A), (C) cleaved caspase-3 and (B), (D) BAX in renal tubular epithelial cells treated with maleic acid compared to those treated with vehicle.
